# Senescence in osteoarthritis: from mechanism to potential treatment

**DOI:** 10.1186/s13075-022-02859-x

**Published:** 2022-07-22

**Authors:** Yikai Liu, Zian Zhang, Tao Li, Hao Xu, Haining Zhang

**Affiliations:** Department of Joint Surgery, the Affiliated Hospital of Qingdao University, Qingdao, 266000 Shandong Province China

**Keywords:** Osteoarthritis, Cellular senescence, Immunotherapy, Killer cells, Natural, SASP (senescence-associated secretory phenotype)

## Abstract

Osteoarthritis (OA) is an age-related cartilage degenerative disease, and chondrocyte senescence has been extensively studied in recent years. Increased numbers of senescent chondrocytes are found in OA cartilage. Selective clearance of senescent chondrocytes in a post-traumatic osteoarthritis (PTOA) mouse model ameliorated OA development, while intraarticular injection of senescent cells induced mouse OA. However, the means and extent to which senescence affects OA remain unclear. Here, we review the latent mechanism of senescence in OA and propose potential therapeutic methods to target OA-related senescence, with an emphasis on immunotherapies. Natural killer (NK) cells participate in the elimination of senescent cells in multiple organs. A relatively comprehensive discussion is presented in that section. Risk factors for OA are ageing, obesity, metabolic disorders and mechanical overload. Determining the relationship between known risk factors and senescence will help elucidate OA pathogenesis and identify optimal treatments.

## Introduction

Osteoarthritis (OA) is the most common joint disease worldwide and imposes substantial mental and physical burdens on elderly individuals. Nearly 250 million people suffer from OA, making it a leading cause of disability in the elderly population [1]. As a degenerative disease, cellular senescence has been proposed to be involved in OA [2]. Cellular senescence is a cell state characterized by permanent cell cycle arrest, resistance to apoptosis, and the continuous secretion of senescence-associated secretory phenotype (SASP) factors [3]. In OA, SASP factors are referred to as a group of inflammatory factors, such as cytokines, chemokines and enzymes. SASP factors are a general name of these inflammatory factors from the perspective of senescence.

Chondrocyte senescence was identified in tissues from human OA patients many years ago, and senescent chondrocytes were shown to accumulate with age in articular cartilage [2]. Recently, there has been renewed interest in studying senescence from a mechanistic angle using OA mouse models, and many senolytic drugs have been studied as a therapeutic approach [4, 5]. Several molecules were found to be pivotal in OA-related senescence, such as GATA [6], STING [7], FOXD1 [8], SIRT6 [9] and DGCR8 [10]. In addition to senescent chondrocytes, senescent fibroblast-like synoviocytes in the osteoarthritic joint also promote OA progression [6]. Synovial fibroblasts, synovial macrophages, osteoblasts and adipocytes are involved in the production of SASPs in addition to chondrocytes during ageing [11]. The inflammatory environment induced by SASP factors is involved in cartilage degeneration and subchondral bone remodelling and eventually leads to cartilage loss and OA progression. Unlike rheumatoid arthritis (RA), OA was first considered a disease associated with wear and tear of joint cartilage rather than inflammation. However, OA has been treated as a combination of injury and inflammation in recent decades, as mounting evidence has revealed the significant role of cytokines and immune cells in the pathology of OA [12–15].

Although the specific mechanism of cellular senescence in OA is unclear, the selective clearance of senescent cells in osteoarthritic mice can attenuate the development of post-traumatic OA (PTOA) [16], while intraarticular injection of senescent cells induced mouse OA [17]. Eliminating p16^INK4A^-positive cells in various organs attenuated age-related deterioration without obvious side effects [18]. The clearance of senescent cells has also been shown to be effective in tau-dependent pathology and cognitive decline-associated diseases [19]. These striking results aroused interest in exploring the underlying mechanism of senescence and its therapeutic targets.

Natural killer (NK) cells play a vital role in the surveillance and killing of senescent cells. Manipulating NK cells to restore the balance between young and senescent cells is promising in age-related diseases. However, whether NK cells function in the senescence environment of osteoarthritic joints remains largely unknown.

The senescent microenvironment in the osteoarthritic joint includes not only senescent chondrocytes, but also synovial fibroblasts and synovial macrophages [11]. Although the senescence of these cells in the joint cavity plays an important role in the pathogenesis of OA, we mainly focused on senescent chondrocytes because chondrocytes are the only cells in cartilage and determine the homeostasis of anabolism and catabolism of the osteoarthritic cartilage matrix. In addition, the senescence of other cells in the joint should never be ignored. In fact, most antisenescence drugs at present are not aimed at a single senescent cell type. Here, we systematically review senescence in OA and propose some ideas for treatment optimization.

## Regulatory factors and characteristics of senescence and the SASP

**Fig. 1:**
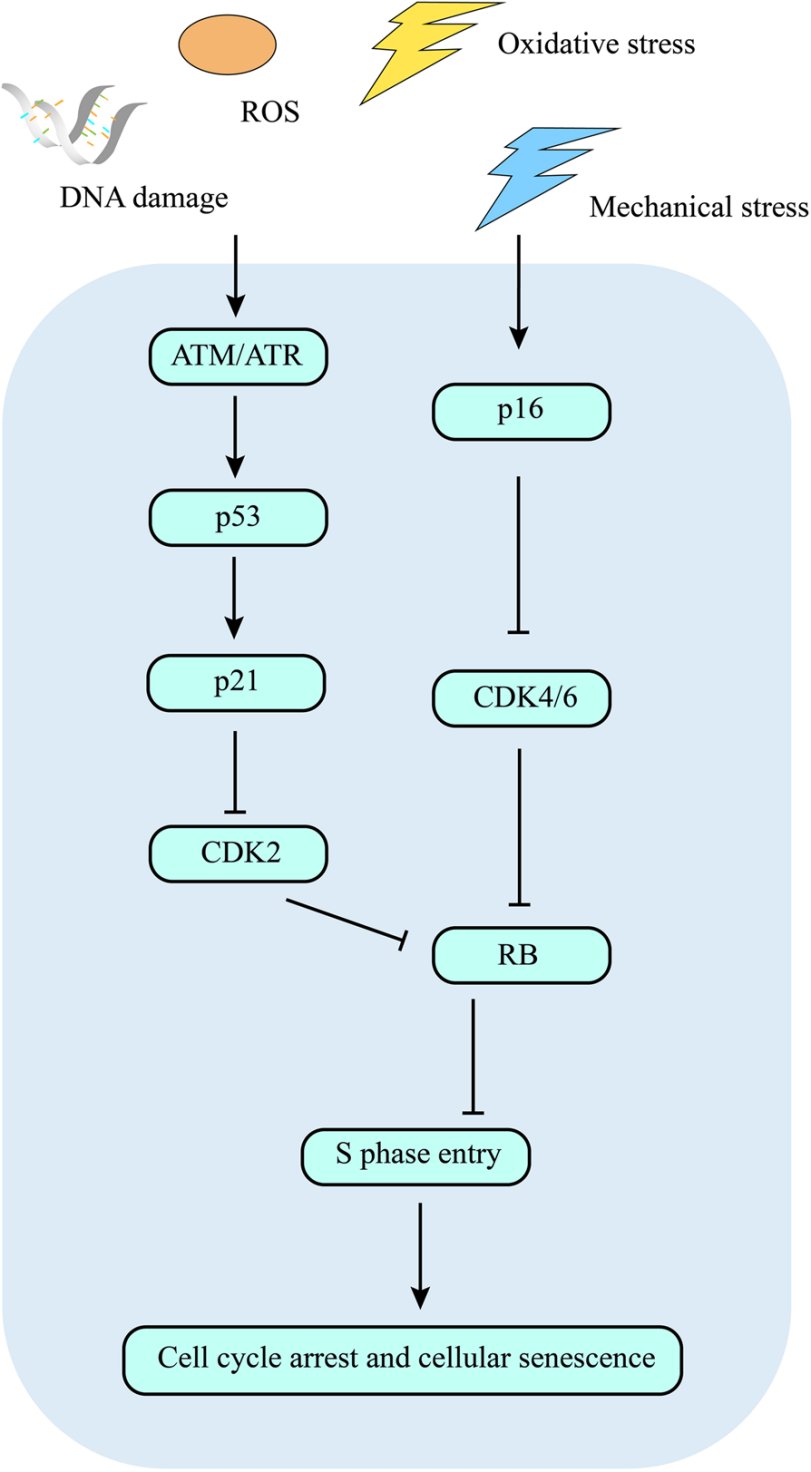
Signalling mechanisms leading to senescence. Mechanical stress, DNA damage, ROS, oxidative stress and other adverse conditions induce cellular senescence. p53-p21-CDK2 and p16-CDK4/6 are two pathways involved in senescence. CDK2/4/6 inhibit RB and promote S phase entry, leading to cell cycle arrest and cellular senescence. ROS, reactive oxygen species; CDK, cyclin-dependent kinases; RB, retinoblastoma protein

The mechanisms leading to senescence are numerous and complicated. DNA damage can induce the DNA damage response (DDR), leading to the direct activation of p53 and its downstream transcription factor p21. Increasing levels of reactive oxygen species (ROS) in the cytoplasm can activate p16, p53 and p21 via the MKK3/6-p38MAPK pathway [20–22]. SASP factors, such as TGF-β and IL-6, can activate p21, p27 and p15 and promote senescence via the SMAD complex or STAT3 pathway, respectively [23–25]. Oncogenic signalling and tumour suppressor inactivation can induce DDR through RAS, Myc and PI3K, resulting in the activation of p53 and p21 [26, 27]. Among these transcription factors, p21 inhibits CDK2, and p16^INK4A^ inhibits CDK4/6 [28, 29]. Then, Rb hypophosphorylation is sustained, and Rb binds with the transcription factors E2F and DP as a result of CDK2/4/6 inhibition. Due to the reduction in E2F and DP, S-phase entry is blocked, followed by cell cycle arrest [4, 21] (Fig. 1).

Although both permanent cell cycle arrest and continuing secretion of SASP factors are characteristics of senescence, these processes have different but overlapping mechanisms. It has been suggested that the production and secretion of SASP factors are independent of cell cycle arrest, as p16^INK4A^ knockout mouse chondrocytes do not decrease the release of SASP factors [30].

The production of SASP factors is associated with NF-κB, p38MAPK and mTOR. SASP can be regulated at different levels from transcription to secretion [31]. The NF-κB pathway is an inflammatory pathway that is involved in the transcription of multiple inflammatory factors [32]. NF-κB is considered a major signalling pathway that stimulates SASP factor production [33]. p38MAPK is induced by stress and is also induced in different types of senescent fibroblasts. p38MAPK inhibition can reduce most SASP factors to a large extent [34]. p38MAPK itself is sufficient to induce the SASP, and this effect is independent of the DDR, promoting long-term SASP factor secretion. Mechanistically, p38MAPK induces SASP by enriching the abundance of SASP factor mRNA and controlling the activity of NF-κB [34]. Rapamycin, an inhibitor of mTOR, can modulate the production of SASP factors by suppressing membrane-bound IL-1α translation and decreasing NF-κB activity [35]. In addition, mTOR regulates the translation of MAPKAPK2, which is also referred to as MK2. MAPKAPK2 phosphorylates and inhibits ZFP36L1, a zinc-finger protein that can degrade the transcripts of SASP factors. Therefore, mTOR is involved in stabilizing SASP components [36].

These SASP factors have upstream and downstream relationships. IL-1α on the cell membrane can enhance the binding ability of NF-κB and C/EBPβ to DNA and then stimulate the transcription of IL-6 and IL-8 [37]. Typical SASP factors, such as IL-6 and IL-8, can enhance SASP factor secretion in turn to form a feed forward loop, thus reinforcing senescence [38].

Early SASP factors, such as TGFβ, can also stimulate normal cells to become senescent cells, leading to the expansion of senescence [39, 40]. Later, SASP factors exert robust abilities to recruit immune cells and exacerbate the inflammatory environment. The switch in SASP is mediated by NOTCH1 [41].

## Possible mechanisms leading to senescence in OA

### Overload and mechanical stress

**Fig. 2:**
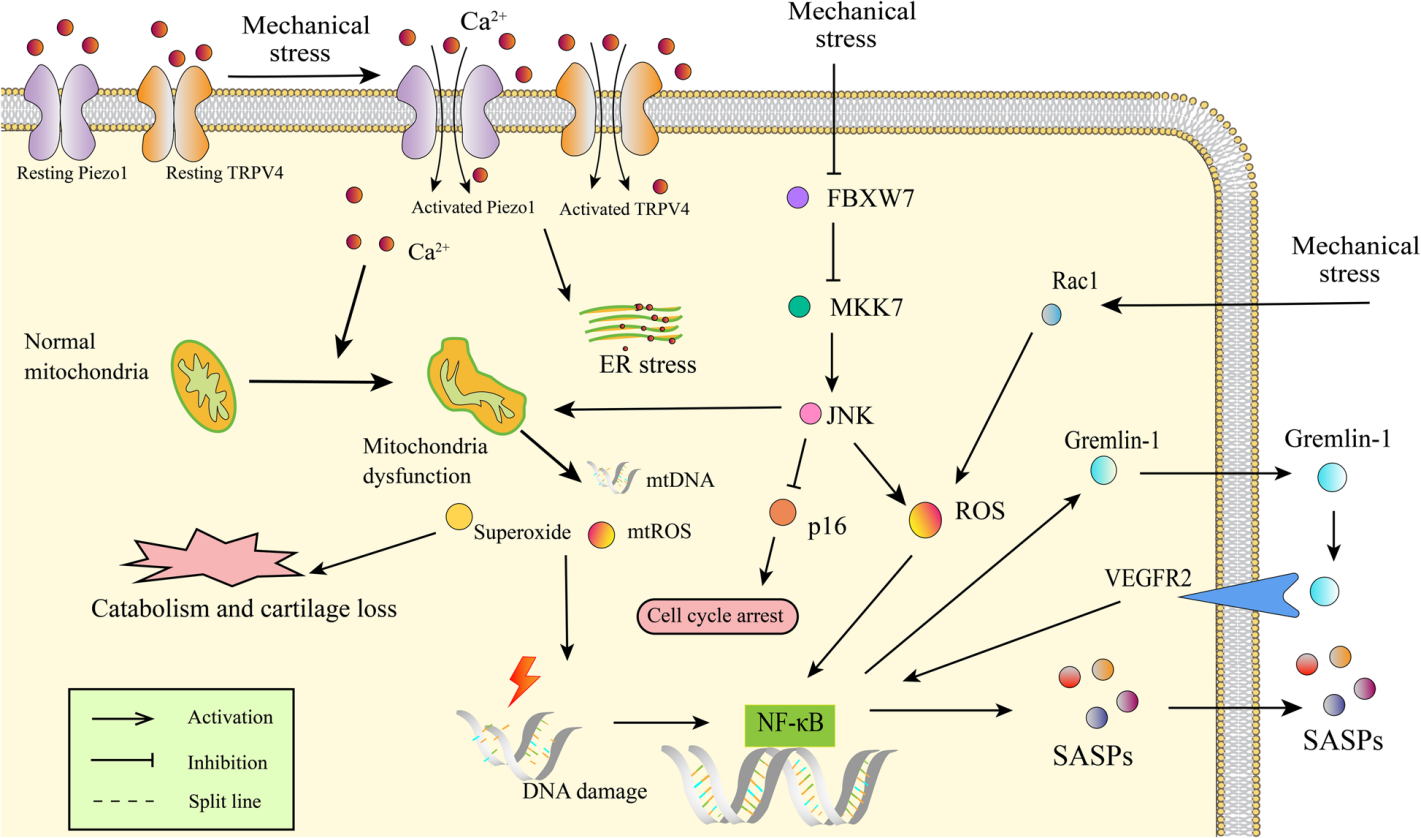
The effects of mechanical stress on chondrocytes. Under mechanical stress conditions, Piezo1 and TRPV4 channels are activated, leading to Ca^2+^ influx into the cytoplasmand triggering endoplasmic reticulum stress and mitochondrial dysfunction. mtROS and mtDNA are released from damaged mitochondria, which results in DNA damage, active catabolism and cartilage degeneration. NF-κB is usually activated during senescence and promotes SASP factor transcription. Mechanical stress inhibits FBXW7-dependent MKK7 degradation, which leads to JNK pathway activation and cellular senescence. JNK also has anti-senescence effect by regulating p16. The Rac1-ROS pathway participates in NF-κB activation under mechanical stress and promotes the production of gremlin-1. Gremlin-1 in turn activates NF-κB via VEGF2 in an autocrine or paracrine manner. TRPV4, transient receptor potential vanilloid 4; mtROS, mitochondrial reactive oxygen species; NF-κB, nuclear factor kappa-B; SASP, senescence-associated secretory phenotype; FBXW7, F-box and WD repeat domain containing 7; MKK7, mitogen-activated protein kinase kinase 7; JNK, mitogen-activated protein kinase; Rac1, Ras-related C3 botulinum toxin substrate 1; VEGF2, vascular endothelial growth factor 2

Mechanical overloading promoted senescence in vitro in cultured chondrocytes and in mouse cartilage [42]. Reduced F-box and WD repeat domain containing 7 (FBXW7) was detected in osteoarthritic patient cartilage, osteoarthritic mouse cartilage, aged mouse cartilage and in vitro cultured primary chondrocytes undergoing mechanical loading. FBXW7 deficiency in chondrocytes induced chondrocyte senescence and promoted cartilage catabolism in mice [42]. Mechanical overloading accelerated chondrocyte senescence by reducing FBXW7 expression and FBXW7-dependent MKK7 degradation, which subsequently stimulated JNK signalling [42]. Inhibition of JNK activity by DTP3 ameliorated chondrocyte senescence and cartilage degeneration, which suggested a harmful effect of JNK activation in osteoarthritic chondrocytes [42]. However, another study suggested JNK deletion enhanced p16 expression in the synovium and cartilage in older mice and promoted age-related OA, indicating that JNK may be a negative senescence regulator in the joint [43]. Mechanical overload is responsible for generating ROS and affects joint degeneration and remodelling [44]. Piezo1 is an ion channel that mediates mechanosensory transduction [45]. Mechanical stretching can induce Ca^2+^ influx via Piezo1, triggering the activation of multiple pathways [46]. The transient receptor potential vanilloid 4 (TRPV4) ion channel is a Ca^2+^ channel, which was first recognized as a transducer of osmotic stress [47, 48]. TRPV4-mediated Ca^2+^ signalling in response to osmotic fluctuations in the cartilage is a potential mechanism by which chondrocytes sense and respond to joint loading [49]. One recent study revealed that a dysfunctional TRPV4-GSK3β pathway prevented osteoarthritic chondrocytes from sensing changes in extracellular matrix viscoelasticity [50]. Excessive mechanical stress induced chondrocyte apoptosis through TRPV4 in an anterior cruciate ligament-transected rat OA model [51]. These findings stressed the importance of TPRV4 in sensing mechanical stress. Other findings indicate that TRPV4 could be a multimodally modulated channel in chondrocytes and synovial cells that interacts with proinflammatory factors and mediates inflammatory signalling and mechanical stress [52, 53]. The activation of Piezo1 and TPRV4 leads to Ca^2+^ influx, Ca^2+^ overload in the cytoplasm, mitochondrial dysfunction and the accumulation of ROS. Then, the DDR occurs, the cell cycle arrests and senescence develops [21, 54]. Moreover, mechanical stress can induce mitochondrial dysfunction through multiple pathways [55]. Gremlin-1 was identified as a mechanical loading-inducible factor in osteoarthritic chondrocytes. A high level of gremlin-1 was detected in middle and deep layers of cartilage after cyclic strain or hydrostatic pressure loading [56]. Mechanistically, gremlin-1 promoted OA progression by activating NF-κB signalling and elevating catabolic enzymes [56] (Fig. 2).

### Inflammatory microenvironment

There is an age-related proinflammatory state termed “ inflamm-aging”. Age-related inflammation can be both systemic and local [57]. Senescent cells produce SASP factors, which promote the inflammatory microenvironment and cartilage catabolism. Chemokines such as CCL2 and CCL4 can recruit macrophages and NK cells [58]. Cytokines such as IL-1, IL-6, IL-7, IL-8, IL-17, OSM, GM-CSF and TNF are involved in OA progression [58–60]. Proteases such as MMP1, MMP3, MMP10, MMP13 and ADAMTS5 can degrade cartilage extracellular matrix (ECM) and promote OA pathology [61, 62]. Growth factors such as TGF-β are involved in the formation of osteophytes [63].

The production of SASPs is not only a characteristic of senescent chondrocytes. Adipocytes, osteoblasts, synovial fibroblasts, synovial macrophages and NK cells in joints are also crucial [11]. In fact, 55% of cytokines are produced by synovial cells, and synoviocyte-chondrocyte interactions play a vital role in the pathogenesis of OA [64].

In addition, senescent cells can release SASPs into their surroundings, which exerts a chemotactic effect on immune cells. The inflammatory microenvironment established by immune cells and the SASP are thought to drive cartilage degeneration by degrading ECM. Few cartilage fragments and other ECM degrading products can induce inflammation. For example, lumican, a major extracellular matrix glycoprotein, is upregulated in osteoarthritic cartilage and synovial fluid (SF). Lumican in SF exacerbates proinflammatory activation induced by TLR4 and leads to macrophage polarization [65].

Moreover, this senescent microenvironment hampers the repair of cartilage by bone marrow stem cells (BMSCs). In vitro coculture assays indicated that senescent chondrocytes and BMSCs interacted with each other. Senescent chondrocytes inhibited proliferation, facilitated senescence, and suppressed chondrogenic differentiation of BMSCs. BMSCs induced the apoptosis of senescent chondrocytes and reduced the proportion of senescent chondrocytes. The intra-articular senescent microenvironment caused by senescent cells and the SASP inhibited the repair of damaged cartilage by BMSCs [66].

### Oxidative stress

Mechanical stress and ageing are two main risk factors for OA, both of which are capable of producing ROS and oxidative stress [44, 67]. Mitochondrial dysfunction and oxidative stress are key factors in OA [67]. mtDNA damage is one possible reason leading to the senescence of osteoarthritic chondrocytes, which may be attributed to increasing mitochondrial ROS production and shortening of telomeres under stress conditions [68]. Poor repair of mtDNA caused by oxidative stress is thought to be associated with OA pathogenesis [69]. ROS promote mitochondrial dysfunction, induce DNA damage and DDR and lead to premature senescence, matrix degradation and subchondral bone mass loss in OA [70]. Oxidative stress results in membrane and nucleic acid damage, as well as the degradation of extracellular components and cartilage destruction. ROS inhibit the synthesis of proteoglycans by NO in the cartilage, and H_2_O_2_ inhibits proteoglycan synthesis by forming ATP and suppressing mitochondrial oxidative phosphorylation [71, 72].

Increased mitochondrial superoxide and decreased SOD2 were induced in chondrocytes under mechanical stress conditions, and the loss of SOD2 led to mitochondrial superoxide accumulation. This imbalance in antioxidants and prooxidants is related to cartilage degeneration [73]. Decreased levels of antioxidant enzymes are associated with accelerated senescence or premature senility, as SOD2^−/−^ mice exhibit decreased lifespans, accelerated ageing cellular phenotypes, and increased p16 and p21 at both the mRNA and protein levels [74]. SOD2 deletion leads to severe OA, while SOD2 overexpression or the use of antioxidants reduces OA [75]. Peroxiredoxins participate in the clearance of excess H_2_O_2_ and act as antioxidants [75, 76]. Under oxidative stress, peroxiredoxins are hyperoxidized and inactivated, which attenuates survival signalling by inhibiting the Akt signalling pathway and promoting p38 signalling in chondrocytes [76]. These studies indicate the negative influences of ROS on cartilage.

Oxidative stress also activates the NF-κB pathway and promotes MMP overproduction, thus triggering DNA damage and cell senescence [77]. Upon exposure to oxidative stress, redox-sensitive PKCδ activates IKKα and triggers its translocation to the nucleus, resulting in phosphorylation and activation p53 [78]. p53 modulates the DDR and orchestrates homeostatic activities and dysfunctional responses, such as irreversible cell cycle arrest and cellular senescence [79].

Inflammation and oxidative stress intensify one other and damage the cartilage [77]. ROS play an important role in the production of SASP components, including IL-1, IL-6 and MMPs [80]. Inflammatory changes decreased the level of antioxidant enzymes in biological fluids and cartilage and increased the level of oxidative agents, which hampered cartilage matrix proteins and led to cartilage damage [77, 81].

### Metabolism and energy shortage

Metabolism is substantially changed and abnormal immunometabolism plays a pivotal role in OA [82]. Obesity is an influential risk factor for OA, and it affects not only the abnormal mechanical stress on joints but also metabolic changes [83, 84]. High body mass index (BMI) was related to hand and knee OA but not hip OA in a 10-year follow-up study [85], which indicates that there must be metabolic abnormalities contributing to OA.

**Fig. 3:**
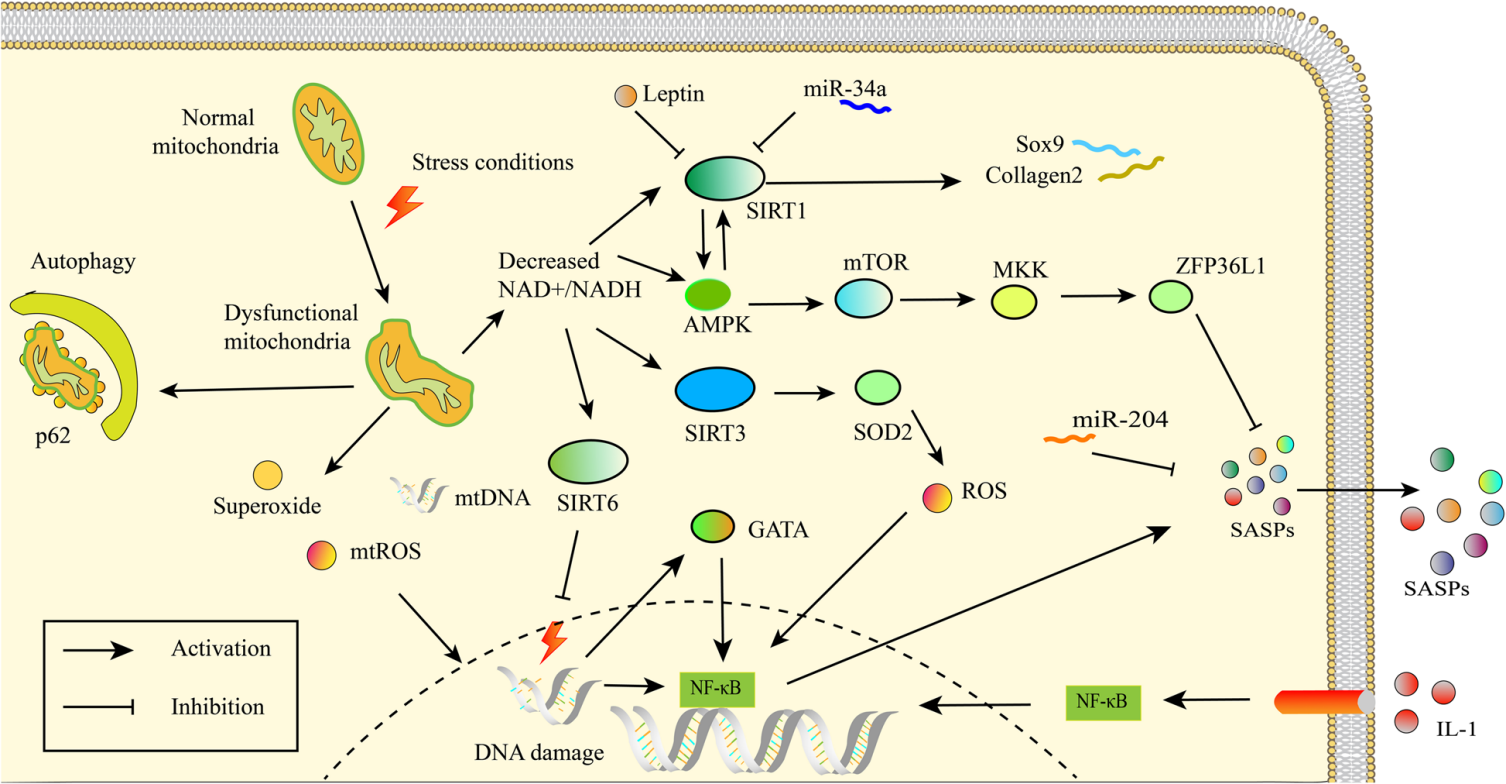
The relationship between energy shortage and chondrocyte senescence. Energy deficiency caused by damaged mitochondria activates AMPK, and SIRTs including SIRT1, SIRT3 and SIRT6. SIRT1 protects cartilage by promoting the transcription of Sox9 and collagen 2. Several factors such miR-34a and leptin inhibit SIRT1 and exacerbate chondrocyte senescence and cartilage damage. Damaged mitochondria are eliminated by p62-mediated autophagy. Activated in an inflammatory environment, NF-κB promotes SASP factor transcription. SASP factors activate NF-κB in an autocrine manner, forming a positive feedback loop. Energy deficiency activates mTOR via AMPK. mTOR inhibits ZFP36L1 by activating MKK. ZFP36L1 and some miRNAs, such as miR-204 participate in the degradation of SASPs. Decreased NAD + /NADH also activates SIRT3 and SIRT6 in addition to SIRT1. SIRT3 deacetylates SOD2 and increases SOD2-specific activity, thus protecting chondrocytes against oxidative stress. SIRT6 can inhibit DNA damage and cellular senescence. SIRT, sirtuin; AMPK, adenosine 5′-monophosphate (AMP)-activated protein kinase; mTOR, mammalian target of rapamycin; ZFP36L1, ZFP36 ring finger protein like 1; SOD2, superoxide dismutase 2

Individuals with obesity show high levels of TNF-α, IL-1β and IL-6 in serum, and these SASP factors are produced by macrophages in adipose tissue [83]. Adipokine and other obesity-associated metabolic factors can induce the expression of proinflammatory cytokines and degradative enzymes, exacerbating cartilage damage and subchondral bone remodelling [84]. Moreover, high levels of adipokines influence M1 macrophage polarization [86], which is considered a proinflammatory phenotype. Free fatty acids (FFAs) increase oxidative stress levels along with increased IL-6 and IL-8 secretion [87]. Leptin induces MMP1, MMP3 and MMP13 expression in chondrocytes [88] and induces chondrocyte senescence by activating the p53/p21 pathways and suppressing sirtuin 1 (SIRT1) [89]. Overall, metabolic syndrome can affect OA development directly by stimulating proinflammatory and catabolic factors and indirectly by interfering with autophagy and senescence (Fig. 3).

Sirtuins (SIRTs) help cells adapt their energy output to match energy requirements by sensing the NAD^+^ concentration in the cytoplasm [90]. The correlation between SIRT and senescence has been verified in many age-related diseases, such as Alzheimer’s disease [91] and diabetes mellitus [92]. SIRTs, especially SIRT1, SIRT3 and SIRT6, exert antiaging effects through various pathways and exhibit connections with mTOR, PI3K, AMPK and NF-κB [93, 94].

In directly cartilage, SIRT1 has been studied most thoroughly, and increasing studies have identified the importance of SIRT6 [95]. SIRT1 conditional knockout mice exhibit accelerated OA progression and are more vulnerable to ageing and mechanical stress stimulation [96, 97]. In addition, SIRT1 interacts with the cartilage-specific transcription factor Sox9 and promoted collagen 2 transcription. SIRT1 and NAD may benefit cartilage by upregulating genes encoding cartilage ECM components [98]. SIRT1 is cleaved to form an inactive N-terminal (NT) polypeptide and a C-terminal (CT) fragment in chondrocytes under proinflammatory stress, and the serum NT/CT SIRT1 ratio is an indicator of early OA and chondrosenescence [99]. Moreover, SIRT1 is related to circadian rhythm, as inhibiting SIRT1 leads to a reduction in the clock gene Bmal1 [100], which is associated with cartilage damage and circadian rhythm disturbances [96] (Fig. 3).

SIRT3 is mainly located in mitochondria and can maintain mitochondrial function, regeneration and dynamics through its deacetylase activity [101]. A recent study proposed that SIRT3-mediated mitochondrial homeostasis protected against OA development [101]. SOD2 is a protective factor against oxidative stress, and SOD2-specific activity decreased with age in mouse chondrocytes due to elevated post-translational lysine acetylation. SOD2 acetylation was also detected in osteoarthritic chondrocytes. SIRT3 is involved in the deacetylation of SOD2, increasing SOD2-specific activity and protecting against OA progression [102] (Fig. 3).

The loss of SIRT6 can increase DNA damage and telomere dysfunction and increase SA-β-Gal-positive chondrocytes and p16 and γH2AX foci, thus inducing premature senescence [9, 103]. SIRT6 overexpression inhibited replicative senescence. Decreased levels of SIRT6 were found in osteoarthritic patient cartilage compared to that of healthy individuals, while overexpression of SIRT6 in a mouse knee OA model retarded OA progression by alleviating the inflammatory response and chondrocyte senescence [104]. IL-1β lowered the expression of SIRT6 and increased the expression of MMP13, and the upregulation of MMP13 could be reversed by overexpressing SIRT6. Further experiments revealed decreased expression of NF-κB but no change in p65 nuclear translocation after SIRT6 overexpression, which established connections between inflammatory pathways and SIRT6 [105]. In addition, these studies indicate that specific activators of SIRT6 may be potential therapeutic targets for controlling senescence and ameliorating OA development (Fig. 3).

### Autophagy

Autophagy usually occurs when cells are under stressful conditions. Damaged and dysfunctional organelles and proteins are engulfed and degraded by lysosomes to save energy under stress conditions, such as nutrient deprivation, hypoxia, ROS and DNA damage [106, 107]. The expression of ULK1, LC3 and Beclin1 in normal human articular cartilage suggests that autophagy is constitutively active in chondrocytes [108], and avascular and hypoxic conditions in cartilage may account for this activation. Autophagy seems to protect chondrocytes against cell death, as both human osteoarthritic chondrocytes and surgery-induced OA mouse models showed decreased ULK1, Beclin1 and LC3 levels and increased apoptosis [108]. In addition, old mice showed reduced autophagic vesicles and decreased levels of ATG-5 and LC3 in knee articular cartilage compared with young mice, with increased levels of the apoptosis marker p85 [109]. Regulated in development and DNA damage response 1 (REDD1), an endogenous inhibitor of mTOR, was protective in the OA model, which suggested the benefit of autophagy in chondrocytes [110]. However, another study noted the dual effects of autophagy on cartilage: in normal human cartilage, autophagy is cytoprotective, and in osteoarthritic cartilage, autophagy promotes death [111].

Autophagy can regulate senescence by controlling protein degradation. Chaperone-mediated autophagy (CMA) accounts for the majority of the lysosome-autophagy proteolytic system [112]. Deficient CMA results in the accumulation of misfolded proteins and oxidative products, and this dysregulation of proteostasis ultimately leads to senescence [112]. In addition, DNA damage promotes senescence by reducing autophagy of GATA4 [113]. The transcription factor GATA4 was identified as a regulator of senescence and the SASP, as it promotes senescence by activating NF-κB and initiating SASP factor production. GATA4 is degraded by p62-mediated selective autophagy under normal conditions, and this process is reduced when senescence occurs [113].

Targeting senescence by controlling autophagy needs further investigation. First, autophagy may partly control some but not all senescence processes. Although rapamycin-mediated inhibition of mTOR prevents SASP induction [36], it fails to prevent or reverse the cell cycle arrest caused by oncogenic RAS [35]. Second, autophagy may be a double-edged sword in senescence; in other words, whether autophagy is beneficial or harmful in osteoarthritic cartilage remains contradictory [114]. Sustained p53 activation occurs even senescent cells are in cell cycle arrest, and activated p53 can induce the transcription of several autophagy-associated proteins such as ULK1, ATG5 and ATG7 [114]. These studies indicated that autophagy may be constitutively activated during senescence. One recent study noted that autophagy leads to senescence via mTORC2 [115]. Autophagic fibroblasts express p16 and p21 and are SA-β-Gal-positive. Suppressing or silencing mTORC2 activation prevents senescent hallmarks [115]. Whether autophagy promotes senescence or protects against senescence should be determined before autophagy-regulating drugs are used to control senescence.

## Therapeutic targeting of senescence

### Drugs that eliminate senescence

Drugs can be roughly divided into two groups: senolytics and senomorphics. The former aims to kill senescent cells, while the latter neutralizes the SASP and offsets its effects. Different molecular targets have been developed.

Senolytics have been proven to improve physical function and increase lifespan in mice [116]. Several senolytics have been developed. Dasatinib targets BCR-ABL, SRC, c-KIT and the ephrin A receptor. Quercetin targets PI3K and serpins. Fisetin targets SIRT1 and IL-1β [117]. UBX0101 targets MDM2, and navitoclax (ABT-263) targets BCL-2 and BCL-XL [118]. Some of these agents are proapoptotic drugs and have been used in cancer treatment. Inducing apoptosis helps to reverse senescence and leads to cell death. Senomorphics, such as lutikizumab and canakinumab, target IL-1β; tocilizumab targets IL-6 receptors, etanercept targets TNF and CL82198 targets MMP13 [4, 63].

Intraarticular injection of quercetin ameliorated cartilage degradation and chondrocyte apoptosis. Reduced intracellular ROS, restored mitochondrial membrane potential and an inhibited Caspase-3 pathway were observed in rat chondrocytes after application of quercetin. In addition, increased M2 polarization, TGF-β and IGF created a pro-chondrogenic microenvironment for cartilage repair [119].

UBX0101 showed encouraging effects in treating an ACLT-induced mouse OA model, regardless of early or later OA stage, in young or old individuals [16]. However, compared to placebo, UBX0101 resulted in no differences in WOMAC-A scores in a 183-person phase II clinical trial of OA (NCT04129944), and a dose–response relationship was not observed. In fact, only the lowest dose showed a curative effect. Fisetin, a flavonoid with potential anti-inflammatory and senolytic functions [117, 120], has been proven to alleviate joint damage in the destabilization of the medial meniscus (DMM) model and is also under investigation in knee OA in a phase I–II clinical trial (NCT 04,210,986).

In addition to senolytic drugs, which aim to kill senescent cells, senomorphic drugs directly inhibit or neutralize SASP factors. IL-1 plays a vital role in cartilage degradation and OA development and has been an attractive target in OA for many years. Mechanistically, IL-1 induces the transcription of MMP13 and ADAMTS5 and is involved in their bioavailability [121]. However, multiple methods to suppress IL-1 did not show satisfactory outcomes in numerous preclinical and clinical studies, including the application of IL-1 receptor antagonist proteins, soluble IL-1 receptors, monoclonal antibodies against IL-1 or against the IL-1 receptor, blocking the formation of active IL-1β, blocking IL-1 signalling pathways and gene therapy [122]. The MMP-13 inhibitor PG-116800 failed in a randomized, double-blind, placebo-controlled, multicentre, parallel-group, dose–response study of treating knee OA, with no clear benefit in relieving pain and improving function [123]. Th17 cells play roles in ankylosing spondylitis, and psoriatic arthritis and various biologic agents, such as IL-17 antibodies, have been developed [124]. One recent study [125] focused on IL-17 and found that a type 17 immune response was induced in joint and draining inguinal lymph nodes after ACLT administration. In vitro experiments demonstrated that Th17 cells induced senescence in fibroblasts. Intraarticular injection of an IL-17-neutralizing antibody in mice alleviated cartilage degeneration and the senescence marker p21 [125]. These results provide evidence of the roles of IL-17 and Th17 cells in OA development and cartilage senescence. However, increased IL-17 mRNA was detected in RA synovial tissue but barely in OA [126]. Whether IL-17 antibodies or other biological agents are useful in humans remains to be tested.

Metformin was recently reported to have effects other than reducing blood sugar, such as attenuating ageing via numerous mechanisms [127]. Activating AMPK and SIRT1, reducing mTORC1, inhibiting NF-κB and inflammatory pathways, regulating the gut microbiota, improving metabolism, prohibiting DNA damage, promoting DNA repair and reducing telomere shortening were involved in the effects of metformin against senescence [127]. One recent study revealed that metformin restricted OA progression through the activation of AMPK signalling [128]. Metformin prevented the formation of osteoclasts, inhibited bone absorption and abnormal subchondral bone remodelling and alleviated OA via the AMPK/NF-κB/ERK signaling pathway [129]. Metformin alleviates monosodium-iodoacetate-induced OA by regulating pain mediators and autophagy [130]. Metformin is promising for controlling senescence and treating OA.

Some senomorphics are under investigation in clinical trials; for examples, tanezumab (NCT02528188) and fasinumab (NCT02683239, NCT03161093, NCT03304379) are NGF inhibitors and are in phase III clinical trials of hip or knee OA. Tocilizumab, an IL-6R inhibitor, is in a phase III clinical trial of hand OA (NCT02477059). The ADAMTS5 inhibitors, GLPG1972/S201086 and M6495, were investigated in phase II clinical trials (NCT03595618) and phase I clinical trials (NCT03583346) of knee OA, respectively. GSK3858279, a CCL17 inhibitor, is in a phase I clinical trial of knee OA (NCT03485365). Otilimab, a GM-CSF inhibitor, is in a phase II clinical trial of hand OA (NCT02683785).

Many questions must be taken into consideration when using senolytics or senomorphics in OA. First, senescence exerts a beneficial effect on wound healing [131, 132] and tumour suppression [133]. Inducing senescence in tumour cells helps the immune system target tumours. The senescent microenvironment produced by senescent cells and the SASP also prohibits tumour progression [134]. Long-term senescent cells may cause chronic inflammation and damage cartilage, but the acute phase of senescence may be useful in ameliorating injury [4, 135]. In addition, there may be off-target effects of senolysis; in other words, these agents do not kill senescent cells specifically. Several chemotherapeutics exhibited effects on total body cells with high proliferative properties. For instance, systemic side effects restrict the use of senolytics for kidney fibrosis [136].

### miRNAs controlling senescence

MicroRNAs (miRNAs) are small noncoding RNAs that can bind to the 3′-untranslated region (3′-UTR) of target mRNAs, inhibit translation and lead to target mRNA degradation. miRNAs play important roles in cellular senescence induced by oxidative stress [137–139]. miRNAs can be a component of exosomes, which are secreted into the ECM and affect other cells in a paracrine manner [138]. Mounting evidence suggests that noncoding RNAs participate in the regulation of SASP factor production at the transcriptional level [140].

As two downstream products of NF-κB, miR-302b and miR-146a exert negative feedback effects on the NF-κB pathway [141]; they inhibit the NF-κB pathway and SASP production by participating in the degradation of the IRAK4 and TRAF6 transcripts, respectively [141, 142]. Notably, miR-140 attenuates early OA development by inhibiting chondrocyte senescence [143]. Insulin-like growth factor 1 receptor (IGF1R) and Toll-like receptor 4 (TLR4) in the PI3K-AKT pathway and JAG1 and NUMBL in the Notch pathway are targets of miR-140 [143]. miR-204 is induced by the transcription factors GATA4 and NF-κB under stress conditions and governs the senescent phenotypes of chondrocytes [144]. miR-204 inhibits various components of proteoglycan (PG) biosynthesis, leading to loss of cartilage and OA development [144]. Inhibiting miR-204 restored PG synthesis, suppressed SASP factor secretion and ameliorated OA in a mouse model [144]. miR-34a affects chondrocyte proliferation, senescence and apoptosis, as reported in another study [145]. miR-34a inhibits SIRT1 expression directly, thus reducing the deacetylation of p53 and promoting senescence, increasing Bax and decreasing Bcl2 [145]. Intraarticular injection of lentivirus containing the anti-miR‑34a sequence slowed the progression of OA [145].

Some miRNAs show antiaging effects in other cells. miR-290 acts as a physiological effector of senescence in mouse embryonic fibroblasts [146]. miR-22 induces cellular senescence and acts as a tumour suppressor by directly targeting CDK6, SIRT1 and Sp1 [147]. Overexpression of miR-302, miR-512-3p and miR-515-3p can rescue Ras(G12V)-induced senescence by inhibiting p21 [148]. miRNAs associated with senescence and OA are listed in Table 1.

**Table 1:** miRNAs involved in senescence or OA

miRNAs	Mainly target molecule or pathways	Effects	References
miR-290		Promotes senescence in mouse embryo fibroblasts	Pitto et al. (2009) [146]
miR-22	CDK6, SIRT1, Sp1 mRNA	Induces cellular senescence and suppresses tumour	Xu et al. (2011) [147]
miR-302, miR-512-3p miR-515-3p	p21 mRNA	Rescue Ras(G12V)-induced senescence	Borgdorff et al. (2010) [148]
miR-302b	IRAK4 mRNA	Inhibits NF-κB pathways	Afonina et al. (2017) [141]
miR-146a	TRAF6 mRNA	Inhibits NF-κB pathways	O'Connell et al. (2012) [142]
miR-34a	SIRT1 mRNA	Inhibits chondrocyte proliferation, promotes senescence and apoptosis	Yan et al. (2016) [145]
miR-204		Inhibits proteoglycan (PG) biosynthesis	Kang et al. (2019) [144]
miR-140	IGF1R and TLR4 in PI3K-AKT pathway and JAG1 and NUMBL in the Notch pathway	Inhibits chondrocyte senescence and attenuates early OA development	Si et al. (2020) [143]
miR-126, miR-130a, miR-142, miR-21, miR-93		Are associated with ageing	Olivieri et al. (2017) [149]

### Immunotherapy against senescence

Compared with drugs that kill senescent cells, the clearance of senescent cells by the immune system seems to be a more selective and more natural method. Some specific epitopes on the cell membrane or the SASP, as well as abnormally expressed proteins, have been found. However, not all of these epitopes are eligible to be treated as targets. For example, p16INK4A, a widely used biomarker of senescent cells, was not suitable as a target, as silencing p16 in adult mouse chondrocytes did not decrease the SASP, nor did it change the rate at which OA occurred in response to physiological ageing or induced joint abrasion. Senescence affects the pathology of OA largely in a SASP-dependent manner, not through the cell cycle arrest itself [30].

Accumulated and activated CD16^+^ NK cells were found in the synovial tissue from patients with active RA, which induced an inflammatory, cytokine-secreting HLA-DR^+^CD90^+^ phenotype of synovial fibroblasts [150]. One study found that CD56^+^/CD16^−^ cells were the major type in the osteoarthritic joint and were correlated with elevated inflammatory factors in SF [151]. Another study revealed that NK cells and neutrophils were the first cells accumulating in the synovium during experimental OA and were involved in the cartilage damage process [152].

**Fig. 4:**
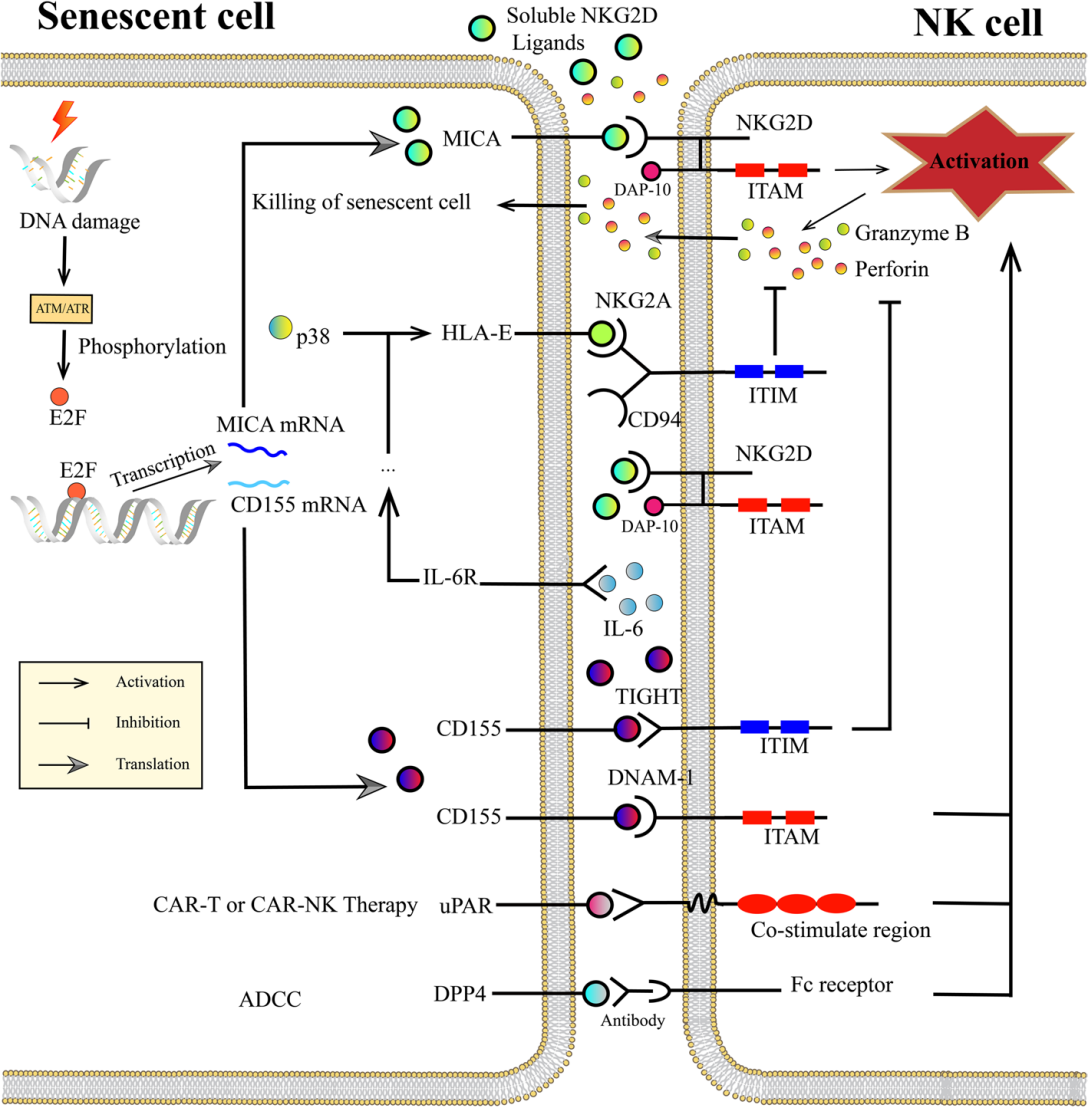
Interactions between NK cells and senescent cells. In response to DNA damage, MICA is selectively expressed on senescent cells rather than proliferative cells. MICA interacts with NKG2D and activates NK cells via ITAM. Activated NK cells produce and secrete granzyme B and perforin to kill senescent cells. Shedding MICA from the senescent cell membrane surface leads to NK cells off-target and senescent cell evasion. HLA-E is upregulated by the p38 pathway. HLA-E binds to NKG2A and inhibits NK cell activation via the ITIM on the NKG2A intracellular segment. CD155 exerts dual effects as its combination with DNAM-1 activates NK cells while its combination with CD94 or TIGHT inhibits NK cell activation. Shedding CD155 participates is involved in the evasion of senescent cells as its binding affinity to DNAM-1 is higher than that to TIGHT and CD94. The expression of MICA and CD155 is directly regulated by transcriptional factor E2F1. uPAR is specifically expressed on the senescent cell membrane surface, and CAR-T therapy targeting uPAR has been designed to eliminate senescence. DPP4 has been treated as a target of immunotherapy via ADCC. NKG2D, natural killer group 2, member D; HLA-E, human leukocyte antigen-E; IL-6, interleukin-6; CAR-T cell, chimeric antigen receptor T cell; uPAR, urokinase-type plasminogen activator receptor; ADCC, antibody-dependent cell-mediated cytotoxicity; DPP4, dipeptide peptidase 4; ITAM, immunoreceptor tyrosine-based activation motif; ITIM, immunoreceptor tyrosine-based inhibitory motif

Senescent human fibroblasts can be identified and killed by NK cells through NKG2D-NKG2D ligand interactions and degranulation of NK cells. NKG2D is expressed on CD4^+^CD28^−^ T cells and NK cells. CD4^+^CD28^−^ T cells rarely appear in healthy individual joint cavities, while these cells accumulate in RA and other autoimmune diseases [153]. RA synovial cells abnormally expressed the stress-inducible MIC ligands of NKG2D, which stimulated CD4^+^CD28^–^ T cell cytokine production and proliferation [153]. However, the expression of Rae1 (a ligand of NKG2D in mice) is induced in all inflammatory joint diseases except collagenase-induced OA (CIOA) mouse models [154], which indicates that the NKG2D-NKG2D ligand interaction is absent in CIOA. It is unclear whether this interaction exists in other types of OA (Fig. 4).

NKG2A acts as an inhibitory receptor on immune cells, which interacts with MHC-I molecules and prevents our own cells from being killed [155]. The balance between activating receptors (such as NKG2D, DNAM-1, NKp44 and NKp46) and inhibitory receptors (such as CD94/NKG2A) determines the killing of senescent synovial cells by NK cells [156]. However, whether NKG2A functions in osteoarthritic joints remains unknown (Fig. 4).

NK cells are particularly suitable for immunotherapy due to their major histocompatibility complex (MHC) and antigen-independent manner [157]. However, the inherent immune clearance of senescent cells could sometimes be in vain due to the shedding of NKG2D ligands [158], low NKG2D ligand expression on senescent cells or low NKG2D expression in NK cells [159]. Some new immunotherapies may help. CD26/dipeptide peptidase 4 (DPP4) was identified as a senescence biomarker, and administration of anti-DPP4 antibody can induce ADCC, leading to the elimination of senescence by NK cells [160]. Several DPP4 inhibitors, such as saxagliptin, teneligliptin and vildagliptin, can ameliorate IL-1β or TNF-α-induced degradation of ECM and protect against chondrocyte senescence [161–163] (Fig. 4).

CAR-T cells or CAR-NK cells are another promising immunotherapy. A chimeric antigen receptor can redirect T cells or NK cells to target specific factors. uPAR is upregulated in chondrocytes during OA and participates in cartilage degradation [164]. In recent studies, urokinase-type plasminogen activator receptor (uPAR) was found to be widely expressed on the senescent cell membrane, and uPAR-specific CAR-T cells were developed to target senescence [165] (Fig. 4).

Many challenges need to be addressed. One limitation of cell therapy is the dense barrier of ECM in cartilage, which prevents immune cells from infiltrating and killing senescent cells. Moreover, OA is a disease with strong heterogeneity and various aetiologies. It would be complicated to identify the specific phenotype before using senolytics or cell therapy. Finally, screening and identifying an epitope of senescent cells in OA is critical. Only when these problems are resolved can immune cell therapy in OA become feasible.

## Conclusions

Many SASP factors have long been known to be proinflammatory producers in the joint cavity or ECM degraders and degeneration catalysts. Several attempts have been made to neutralize the SASP but have not shown satisfactory outcomes in OA treatment. The senolytic drug UBX0101 failed to outperform the placebo in a phase II clinical trial. One possible reason for this failure to translate treatment into clinical use is the widely used 8-week-old mouse model rather than the aged mouse model which may reflect and guide application more robustly. Immunotherapy in OA is rather new and shows potential, as it is naturally in accordance with establishing normal homeostasis in old and young cells. Therapeutic molecular targets that can utilize the immune system need to be developed.

## Data Availability

Data sharing is not applicable to this article as no datasets were generated or analysed during the current study.

## Abbreviations

OA: Osteoarthritis
RA: Rheumatoid arthritis
SASP: Senescence-associated secretory phenotype
PTOA: Posttraumatic osteoarthritis
NK cells: Natural killer cells
DDR: DNA damage response
ROS: Reactive oxygen species
MKK: Mitogen-activated protein kinase kinase
p38MAPK: P38 mitogen-activated protein kinase
IL-6: Interleukin-6
TGF-β: Transforming growth factor-β
PI3K: Phosphoinositide 3-kinase
CDK: Cyclin-dependent kinases
RB: Retinoblastoma protein
NF-κB: Nuclear factor kappa-B
mTOR: Mammalian target of rapamycin
CCL: Chemokine (C–C motif) ligand
MMP: Matrix metalloproteinase
TRPV4: Transient receptor potential vanilloid 4
FBXW7: F-box and WD repeat domain containing 7
MKK7: Mitogen-activated protein kinase kinase 7
JNK: Mitogen-activated protein kinase
Rac1: Ras-related C3 botulinum toxin substrate 1
VEGF2: Vascular endothelial growth factor 2
ECM: Extracellular matrix
ADAMTS5: A disintegrin and metalloproteinase with thrombospondin motifs 5
SF: Synovial fluid
TLR4: Toll-like receptor 4
BMSCs: Bone marrow stem cells
mtDNA: Mitochondrial DNA
SOD2: Superoxide dismutase 2
BMI: Body mass index
TNF-α: Tumour necrosis factor-α
FFAs: Free fatty acids
SIRT: Sirtuin
HDACs: Histone deacetylases
NAD: Nicotinamide adenine dinucleotide
PAI-1: Plasminogen activator Inhibitor-1
PCSK9: Proprotein convertase subtilisin/kexin 9
AMPK: Adenosine 5′-monophosphate (AMP)-activated protein kinase
REDD1: DNA damage response 1
CMA: Chaperone-mediated autophagy
ATM: Ataxia telangiectasia mutated
ATR: Ataxia telangiectasia and RAD3-related protein
3’-UTR: 3′-Untranslated region
IRAK4: Interleukin-1 receptor-associated kinase 4
TRAF6: TNF receptor-associated factor 6
PG: Proteoglycan
IGF1R: Insulin-like growth factor 1 receptor
ACLT: Anterior cruciate ligament transaction
WOMAC: Western Ontario and McMaster Universities Osteoarthritis Index
ADCC: Antibody-dependent cell-mediated cytotoxicity
DPP4: Dipeptide peptidase 4
CAR-T cell: Chimeric antigen receptor T cell
CAR-NK cell: Chimeric antigen receptor NK cell
NKG2D: Natural killer group 2, member D
HLA-E: Human leukocyte antigen-E
SLE: Systemic lupus erythematosus
CIOA: Collagenase-induced osteoarthritis
uPAR: Urokinase-type plasminogen activator receptor
SIRPα: Signal-regulatory protein alpha
